# Nuclear processing of nascent transcripts determines synthesis of full-length proteins and antigenic peptides

**DOI:** 10.1093/nar/gky1296

**Published:** 2019-01-09

**Authors:** Rodrigo Prado Martins, Laurence Malbert-Colas, María José Lista, Chrysoula Daskalogianni, Sebastien Apcher, Marika Pla, Sarah Findakly, Marc Blondel, Robin Fåhraeus

**Affiliations:** 1 Université Paris 7, Inserm, UMR 1162, Paris, France; 2 Université de Brest, Inserm, EFS, UMR 1078, GGB, F-29200 Brest, France; 3 ICCVS, University of Gdańsk, Science, ul. Wita Stwosza 63, 80-308 Gdańsk, Poland; 4 Institut Gustave Roussy, Université Paris Sud, UMR 1015, Villejuif, France; 5 Université Paris 7, IUH, Inserm, UMR-S-1131, Paris, France; 6 Department of Medical Biosciences, Umeå University, Umeå, Sweden; 7 RECAMO, Masaryk Memorial Cancer Institute, Zluty kopec 7, 656 53 Brno, Czech Republic

## Abstract

Peptides presented on major histocompatibility (MHC) class I molecules form an essential part of the immune system's capacity to detect virus-infected or transformed cells. Earlier works have shown that pioneer translation peptides (PTPs) for the MHC class I pathway are as efficiently produced from introns as from exons, or from mRNAs targeted for the nonsense-mediated decay pathway. The production of PTPs is a target for viral immune evasion but the underlying molecular mechanisms that govern this non-canonical translation are unknown. Here, we have used different approaches to show how events taking place on the nascent transcript control the synthesis of PTPs and full-length proteins. By controlling the subcellular interaction between the G-quadruplex structure (G4) of a gly-ala encoding mRNA and nucleolin (NCL) and by interfering with mRNA maturation using multiple approaches, we demonstrate that antigenic peptides derive from a nuclear non-canonical translation event that is independently regulated from the synthesis of full-length proteins. Moreover, we show that G4 are exploited to control mRNA localization and translation by distinguishable mechanisms that are targets for viral immune evasion.

## INTRODUCTION

Viral interference with the host cell provides windows of opportunities to disclose cell biological processes. Viral evasion of the immune system is no exception and the different steps of antigen presentation on major histocompatibility (MHC) class I molecules have largely been characterized using viral factors (1). Previous works by our and other teams have focused on how the Epstein-Barr virus (EBV)-encoded EBNA1 employs a *cis*-acting mechanism to suppress translation of its own mRNA in order to minimize the production of antigenic peptide substrates and thereby avoid host immune response (2,3).

Full-length proteins are poor substrates for the MHC class I pathway, at least in experimental conditions, and alternative sources of antigenic peptide substrates have been suggested (4–6). Previous works have shown little difference in antigen presentation if the antigenic peptide is derived from introns or exons, from 3′ UTRs or from mRNAs targeted for nonsense-mediated decay (NMD) (7–9). Transfected capped ovalbumin (OVA) mRNAs produce full-length proteins as long as the mRNA is present for at least eight hours, whereas antigenic peptide substrates from the same mRNAs are synthesized up to 2 h post-transfection (7). Furthermore, intron-derived nascent peptides can be detected in the nuclear compartment (8). These different observations implicate a non-canonical nuclear mRNA translation event producing pioneer translation products (PTPs) for the MHC class I pathway.

Canonical mRNA translation is initiated at the m^7^Cap-structure by the binding of the eIF4E, which normally leads to translation initiation at the first downstream AUG codon (10). Alternatively, the ribosome can bypass the m^7^Cap and initiate directly on the message via internal ribosome entry sites (IRES) that are normally located in the 5′ UTRs (11,12). The requirement for initiating at AUG is not absolute and it has been proposed that a large amount of small peptide products detected by mass spectrometry are initiated from non-AUG codons (13).

The newly synthesized pre-mRNAs engage with the splicing machinery and with RNA quality control mechanisms. Following splicing, mRNAs bind nuclear export factors such as the export factor 1 (NFX1) for transport through the nuclear pore complex (14). However, some cellular and viral RNAs, like those exported by the HIV-1 Rev machinery, instead use the chromosome region maintenance 1 protein homologue (CRM1) for cytoplasmic transport (15). So far, little is known of how alternative mRNA processing pathways affect nuclear export and translation.

Nucleolin (NCL) is a multifunctional protein that interacts with RNAs, DNAs and proteins. It plays a major role in ribosomal biogenesis but is also implicated in various processes such as replication, transcription, cell cycle control and apoptosis (16). As an RNA binding protein (RBP), NCL displays a preference for endogenous and exogenous G-rich sequences, including G-quadruplexes (G4), found in transcripts untranslated and coding regions (17,18). This protein is reported to stabilize G4 structures in viruses such as HIV (19) and in mRNAs encoding cancer proteins (20). A *cis*-mediated translation control of EBNA1 involves NCL binding to the G4 structure formed by the gly-ala repeat (GAr)-encoding sequence in EBNA1 transcript. Consequently, NCL binding is necessary for the GAr-mediated inhibition of antigen presentation (21,22). G4 RNA structures are also implicated in controlling gene regulation by affecting splicing, polyadenylation, mRNA translation and stability (20,23–25).

In this study, we took advantage of the unique properties of the NCL-GAr mRNA interactions to control translation and used different tools as intronless and intron-bearing constructs, IRES structures and alternative RNA export to show that events taking place on nascent transcripts determine the synthesis of neoantigens and full-length proteins. These results further demonstrate the existence of different mRNA translation events for the production of peptide products with alternative functions and will help to design future vaccine strategies against virus-infected or transformed cells.

## MATERIALS AND METHODS

### Plasmid constructions

**Figure 1.**
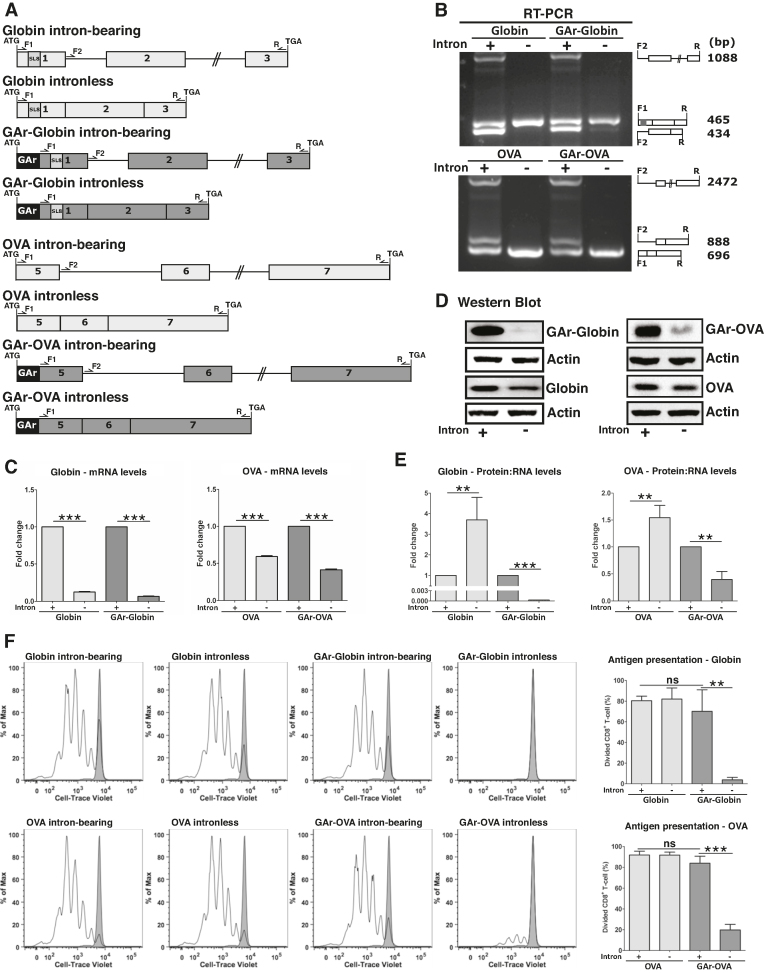
mRNA splicing prevents GAr-mediated suppression of canonical translation and antigen presentation. (**A**) Cartoon illustrating Globin and Ovalbumin (OVA) constructs. Intron-bearing and intronless constructs were generated with, or without, the coding sequence of the gly-ala repeat (GAr) in their 5′ end. OVA-derived SIINFEKL antigenic peptide (SL8) was inserted into the exon 1 of Globin constructs. (**B**) RT-PCR from Globin (upper panel) and OVA (lower panel) mRNAs. cDNA from indicated constructs was analyzed by multiplex PCR using F1, F2 and R primers (represented in A), revealing that the GAr does not affect splicing. Cartoons (right) represent primers and the corresponding RT-PCR products. (**C**) Graphs show the relative mRNA levels by qPCR from cells transfected with indicated constructs. (**D**) Western blots show that the GAr-mediated translation suppression is disrupted in intron-bearing mRNAs. Intron-bearing and intronless constructs without the GAr show similar protein levels. One of three independent experiments using Globin (left) and OVA (right) constructs is shown. (**E**) Data from (C) and (D) were used to calculate the relative protein:mRNA ratios. mRNAs from intronless constructs without the GAr are more efficiently translated than corresponding intron-bearing constructs except for GAr-carrying constructs. Graphs show the mean of intronless:intron-bearing ratios from three independent experiments. (**F**) H1299 cells transiently expressing murine MHC-I (Kb) and the indicated constructs were co-incubated with OT-1 CD8^+^ T-cells labelled with Cell-trace Violet. The levels of OT-1 cells proliferation were analyzed by FACS. Open peaks in the histogram represent successive generations of T-cells indicating T cell activation. Grey peaks denote unstimulated controls. Cells expressing GAr-intronless constructs failed to stimulate OT-1 cells. Graphs (right) depict the mean percentage of dividing CD8^+^ T-cell from three independent experiments. **P< 0.01, ***P< 0.001, ns: not significant.

The sets of splicing reporters described in Figure 1 were constructed as described in Supplementary Figure S1. Briefly, full-length Globin intron-bearing and OVA exon 5–7 fragment were amplified by PCR from the Glob-exon-SL8 construct (8) and chicken genomic DNA, respectively. The amplicons were cloned into pcDNA3 to create the control constructs or replaced the OVA ORF of the pcDNA3-GAr-OVA construct (26) to generate plasmids with the GAr domain upstream and in the same frame of the inserts. Afterwards, RNA from H1299 cells transfected with the resulting intron-bearing constructs was used to produce cDNA employing M-MLV reverse transcriptase and oligo-dT (Invitrogen). The resulting cDNAs were employed in a second round of cloning using the same primers and pcDNA3 or pcDNA3-GAr-OVA construct as vectors to produce the corresponding intronless constructs. Globin intronless-RRE, GAr-Globin-intron-bearing-RRE and GAr-Globin intronless-RRE constructs were cloned following the same method using the plasmid Glob-exon-SL8-RRE (8), herein Globin intron-bearing-RRE, as template. C-myc-IRES-OVA construct was described elsewhere (2) and for generating HCV-IRES-OVA construct, IRES was PCR amplified from HCV genomic DNA and cloned into the 5′ UTR of pcDNA3-OVA (2). The HA-NCLΔNLS plasmid was created by replacing the NLS sequence KKRA of a pcDNA3-HA-NCL construct (21) with AARA by site-directed mutagenesis. The Rev expression vector was a gift from Ali Saïb, Saint-Louis Hospital, Paris. All constructs were amplified in DH5α *Escherichia coli* and sequenced by Sanger method. The list of plasmids, primers and restriction enzymes used in this study is provided in Supplementary Table S1.

### Cell culture, transfection and drug treatments

The human lung carcinoma cell line H1299 was cultured in RPMI-1640 supplemented with 10% fetal bovine serum (FBS), 2 mM l-glutamine, 100 U/ml penicillin and 100 μg/ml streptomycin. Transient transfections were performed using Genejuice reagent (Merck Bioscience) according to manufacturer's protocol. All cells were cultured at 37°C with 5% CO_2_. For cell treatments with isoginkgetin (Sigma) and PhenDC3 (27), cells were incubated with 30 and 5 μM of drug for 24 and 40 h after transfection, respectively. Drug stock solutions were prepared in DMSO (Euromedex).

### RNA extraction, RT-PCR and qRT-PCR

H1299 cells were plated in six-well plates and transfected with the indicated constructs. At 48 h post transfection, cells were washed with cold PBS and RNA was purified using the RNeasy Mini Kit and on-column DNase treatment (Qiagen) following the manufacturer's protocol. Nuclear/cytoplasmic RNA extractions were carried out as described elsewhere (8) employing transfected H1299 cells. RT was carried out using M-MLV reverse transcriptase and random hexamers or oligo(dT) primers (Invitrogen). RT-PCR was performed using Phusion high-fidelity PCR master mix (Thermo Scientific) and a set of primers described in Figure 1A and Supplementary Table S2. qRT-PCR was performed as previously described (8) using specific primer pairs for each gene fragment of interest (see Supplementary Table S2).

### Western blot analysis

Whole cell lysates were prepared 48 h post-transfection and protein concentration was measured using a Bradford assay. Samples were electrophoretically separated in NuPAGE^®^ Bis-Tris gels 10% (Invitrogen), transferred onto 0.45 μm nitrocellulose membranes (GE) and blotted under standard conditions using the following antibodies: mouse monoclonal anti-HBB antibody (2H3, Sigma), rabbit polyclonal anti-OVA antibody (C6534 Sigma), mouse monoclonal anti-actin (AC-15 Sigma), mouse polyclonal anti-HA antibody (a kind gift from Borek Vojtesek, Masaryk Memorial Cancer Institute, Brno, Czech Republic) and chicken polyclonal anti-HIV-1 Rev (ab36623, Abcam). Anti-mouse (Dako), anti-rabbit (Dako) or anti-chicken (Sigma) secondary antibodies conjugated to horseradish peroxidase were used to generate immunocomplexes revealed with enhanced chemiluminescence (Thermo Scientific). Membranes were scanned in a MyECL imager (Thermo Scientific) and signal intensity was determined using My Image software (Thermo Scientific).

### Immunofluorescence and RNA-FISH

H1299 cells were plated on 12-mm-diameter coverslips in 24-well plates, and transfected with the indicated constructs or pCDNA3. At 24 h post-transfections cells were fixed with 4% paraformaldehyde for 20 min, permeabilized with PBS 0.4% Triton X-100, 0.05% CHAPS for 10 min at room temperature and saturated with PBS 3% BSA for 30 min. Samples were incubated overnight at 4°C with rabbit polyclonal antibody anti-NCL (ab22758, Abcam) or mouse monoclonal anti-HA-Tag conjugated to Alexa Fluor^®^ 488 (6E2, Cell Signaling). A goat anti-rabbit Ig antibody conjugated to Alexa Fluor^®^ 647 (Sigma) was used as secondary antibody when necessary. RNA-FISH assays were performed employing Globin and OVA probes obtained from Biosearch Technologies according to the manufacturer's protocol. For nucleolar co-staining, samples were incubated with anti-nucleolin conjugated to Alexa Fluor^®^ 488 (ab154028, Abcam) after probes hybridization. For immunofluorescence coupled to RNA-FISH, samples were incubated with chicken polyclonal anti-HIV-1 Rev antibody (ab36623, Abcam) after hybridization and immunocomplexes were detected under standard conditions using goat anti-chicken IgY conjugated to Alexa Fluor^®^ 568 (Abcam). Samples were examined in an LSM 800 confocal laser microscope (Carl Zeiss MicroImaging GmbH, Jena, Germany).

### Proximity ligation assay (PLA) for mRNA - protein interactions

H1299 cells were grown, fixed and permeabilized as described for immunofluorescence. Samples were overnight hybridized with 50 ng of a Globin DNA probe (5′-GGCCTCACCACCAACTTCATCCACGTTCACCTTGCAAAAA-3′) conjugated to digoxigenin in the 3′ end. Afterwards, samples were saturated with PBS 3% BSA, 0.1% saponine and incubated for 2 h with mouse monoclonal anti-digoxigenin (DI-22, Sigma) and rabbit polyclonal anti-NCL (ab22758, Abcam) primary antibodies diluted in blocking solution. The proximity ligation assay (PLA) was carried out using the Duolink PLA *in situ* kit (Sigma) following the manufacturer's protocol. For co-staining of HIV-1 Rev protein in PLA samples, chicken polyclonal anti-HIV-1 Rev antibody (ab36623, Abcam) and goat anti-chicken IgY conjugated to Alexa Fluor^®^ 568 (Abcam) were added to primary antibodies and Duolink secondary antibodies mixes, respectively. For co-staining of NCLΔNLS or nucleolus, samples were incubated for 45 min with mouse monoclonal anti-HA-Tag antibody conjugated to Alexa Fluor^®^ 488 (6E2, Cell Signaling) or anti-Fibrillarin antibody conjugated to Alexa Fluor^®^ 488 (EPR10823B, Abcam) after the PLA amplification step.

### *In vivo* and *in vitro* protein–RNA co-immunoprecipitation

*In vivo* protein–RNA co-immunoprecipitation was carried out as described elsewhere (28) using a rabbit polyclonal anti-NCL (ab22758, Abcam). For *in vitro* assays, 1 μg of total RNA from transfected cells were co-incubated under agitation with 100 ng of recombinant NCL (MyBioSource) in binding buffer (50 mM Tris pH 7.5, 150 mM NaCl, 0.02 mg/ml yeast tRNA, 0.2 mg/ml BSA) at 4°C. After incubation, NCL–RNA complexes were pulled down using protein B-coated sepharose beads according to standard conditions and purified using the TRIzol (Life Technologies). Precipitated RNAs were analyzed by RT-qPCR using the primers pairs described in the Supplementary Table S2.

### Antigen presentation assays

Naive OVA_257–264_ specific CD8^+^ T cells were isolated by negative selection from peripheral and mesenteric lymph-nodes of 12 weeks old female OT-I mice using the CD8^+^ T cell isolation kit (Miltenyi Biotec, Germany). Afterwards, CD8^+^ T cells were stained with CellTrace™ Violet (Thermo Fisher Scientific, USA) according to the manufacturer's protocol and mixed with H1299 cells co-transfected with mouse Kb expression vector and the indicated constructs. For all the assays, 10^5^ H1299 cells were harvested 48 h after transfection or after treatment and co-incubated with 4 × 10^5^ stained CD8^+^ T cells at 37°C in humidified air/CO_2_ atmosphere in RPMI medium containing 10% FBS, 4 mM l-glutamine, 100 U/ml penicillin, 100 μg/ml streptomycin, 5 mM HEPES and 0.05 mM 2-mercaptoethanol (Sigma-Aldrich). After 3 days, cells were harvested, stained with hamster anti-mouse CD3-APC (Miltenyi Biotec) and fixable viability dye eFluor^®^ 780 (eBioscience, USA) and analyzed on a CANTO II flow cytometer (BD Biosciences, USA). Cells were gated for live CD3^+^ cells (10 000 events collected) and data were analyzed using FlowJo software version 8 (Tree Star). The percentage of proliferating T cells was considered for statistical analysis (29). For IL-2 release analysis, supernatants were collected after 3 days of co-incubation and IL-2 levels were measured employing the IL-2 ELISA MAX™ Standard kit (Biolegend, USA) according to manufacturer's instructions.

### Polysome profiling

Five–fifty percent (w/v) linear sucrose gradients were freshly casted on SW41 ultracentrifuge tubes (Beckmann) using the Gradient master (BioComp instruments) following the manufacturer's instructions. Forty-eight hours post transfection, H1299 cells (with 80% confluency) were treated with cycloheximide 100 μg/ml for 5 min at 37°C and washed twice with 1× PBS (Dulbecco modified PBS, GIBCO) containing cycloheximide 100 μg/ml. Cells were then scraped and lysed with polysome lysis buffer (100 mM KCl, 50 mM HEPES–KOH, 5 mM MgCl_2_, 0.1% NP-40, 1 mM DTT, cycloheximide 100 μg/ml, pH 7.4). Lysates were loaded on a sucrose gradient and centrifuged at 222 228 × g for 2 h at 4°C in a SW41 rotor. Samples were fractionated using Foxy R1 fraction collector (Teledyne ISCO) at 0.5 min intervals (30). RNA purifications from fractions were performed using ethanol precipitation combined with RNeasy Mini Kit (Qiagen). RT and qRT-PCR were performed as described above using primers described in Supplementary Table S2. The relative distribution of target mRNA was calculated using fraction 1 as reference according to Panda *et al.* (31).

### Statistical analysis

Data were analyzed by ANOVA in conjunction with Tukey's test or two-tailed unpaired Student's t-test using GraphPad Prism 5 for Windows (GraphPad Software). Data shown are mean ± s.d. of minimum three independent experiments. *P < 0.05; **P < 0.01; ***P < 0.001; ns, not significant.

## RESULTS

### A gly-ala repeat (GAr) of EBNA1 suppresses mRNA translation and antigen presentation in the context of intronless, but not intron-bearing, sequences

Accumulating evidence show that antigenic peptides for the MHC class I pathway are derived from alternative mRNA translation products (2,7,8). To better understand the processes producing antigenic peptide substrates, we took advantage of the unique properties of a gly-ala (GAr)-encoding sequence that, when fused to the 5′ coding sequence of any mRNA, suppresses translation *in cis* and impedes the production of antigenic peptide substrates for the MHC class I pathway (26,32). We set up different strategies to modify the way GAr-carrying mRNAs are processed, trafficked and interact with specific proteins and how this affects the synthesis of full-length proteins and antigenic peptide substrates. We first created two sets of constructs in which mRNAs encoding β-Globin (Globin) or chicken ovalbumin (OVA) are expressed *via* splicing of intron-bearing sequences (Globin intron-bearing and OVA intron-bearing) or from intronless sequences (Globin intronless and OVA intronless). The same constructs were further used to include the GAr-encoding sequence in their 5′ coding sequences (GAr-Globin intron-bearing, GAr-OVA intron-bearing, GAr-Globin intronless and GAr-OVA intronless). Subsequently, the SL8 epitope from chicken OVA was inserted into the first exon of Globin constructs (Figure 1A and Supplementary Figure S1).

Using the indicated forward (F) and reverse (R) primers and RT-PCR we could confirm that the presence of the SL8 epitope, or the GAr, did not affect pre-mRNA splicing and that the mRNAs derived from spliced or intronless sequences are the same (Figure 1B). In line with previous reports (33), intron-bearing sequences gave rise to higher mRNA levels, as compared to the corresponding intronless messages, independently of the presence of the GAr (Figure 1C). Interestingly, the presence of introns overcame the translation inhibitory effect of the GAr in both the OVA and the Globin context (Figure 1D). Estimated protein:RNA ratios indicated that intron-bearing mRNAs are less efficiently translated than intronless transcripts, except for the GAr-carrying constructs (Figure 1E). Of note, polysome fractionation of cells expressing globin intron-bearing or intronless constructs also showed that mRNAs from intronless sequences are more prevalent in heavy polysomes than their counterparts from intron-bearing sequences, indicating that the former are more efficiently translated. Furthermore, the analysis of polysome fractions also revealed a higher accumulation of GAr-intronless mRNA in light ribosomes, as compared to GAr-carrying mRNAs from intron-bearing sequences. This observation further confirms that the GAr-mediated inhibition of mRNA translation is disrupted in the context of intron-bearing sequences (Supplementary Figure S2A and B).

We next tested if the effect of introns on preventing GAr-mediated suppression of protein synthesis also affected its capacity to inhibit the production of antigenic peptide substrates. OT-1 cells are TCR transgenic CD8^+^ T lymphocytes from the OT-1 mice, which specifically target OVA SL8 epitopes presented on murine MHC class I molecules (Kb). Cell-trace Violet-labelled OT-1 cells were incubated with H1299 cells expressing the Kb and the indicated SL8-encoding constructs. Production of antigenic peptide substrates was estimated by determining OT-1 cell proliferation using FACS analysis (Figure 1F) (29). In both the context of OVA and Globin, we observed that the GAr suppressed the production of antigenic peptide substrates only in the context of intronless, and not intron-bearing, mRNAs.

### Splicing alters GAr mRNA trafficking and prevents the NCL – GAr mRNA interaction

**Figure 2.**
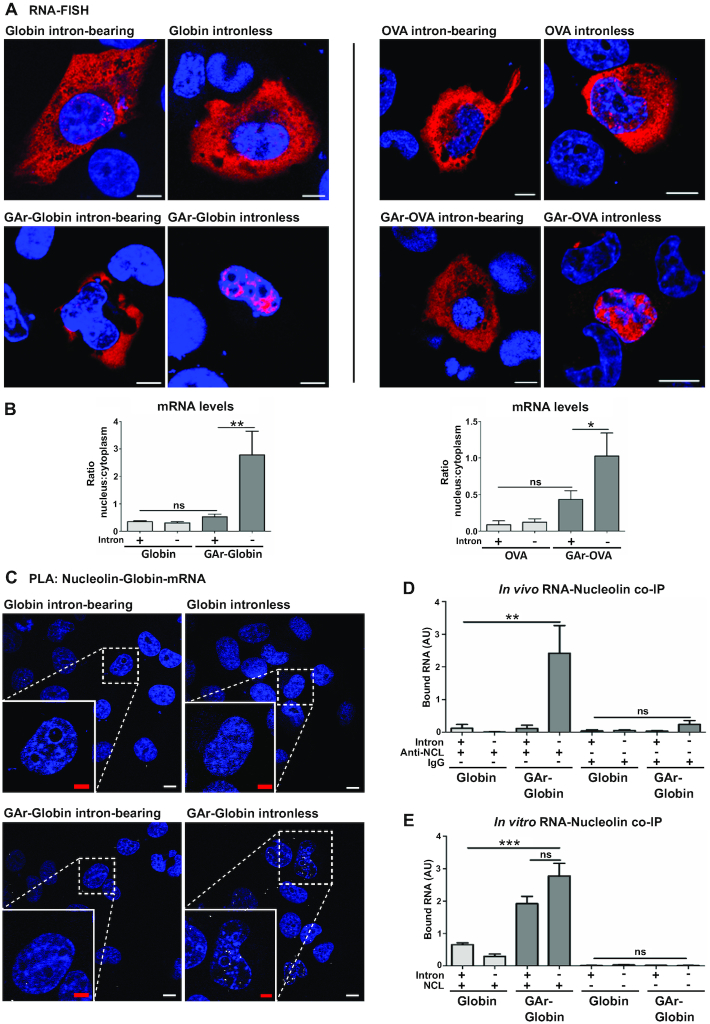
Splicing modifies mRNA localization and prevents nucleolin (NCL) - GAr mRNA interaction. (**A**) Cells expressing the indicated constructs were analyzed by RNA-FISH using probes against Globin or OVA mRNA. Fusion of the GAr to intronless Globin or OVA shifts the localization of mRNA from predominantly cytoplasmic to predominantly nuclear. The GAr does not affect export of corresponding intron-bearing constructs. Red and blue represent mRNAs and DAPI, respectively (see also Supplementary Figure S3). Scale bar = 10 μm. (**B**) Graphs show the relative ratio of nuclear to cytoplasmic mRNA levels from cells as in (A) using RT-qPCR. Histograms represent the mean of three independent experiments. *P< 0.05, **P< 0.01, ns: not significant. (**C**) Proximity Ligation Assay (PLA) showing the interaction between NCL and GAr-carrying mRNAs. PLA complexes are depicted as white dots and blue represents DAPI (for PLA controls see Supplementary Figure S4). NCL interacts with the G4 structure of the GAr-Globin intronless mRNA (lower right) predominantly in the nucleus. No interactions are observed in cells transfected with the GAr-Globin intron-bearing or constructs without the GAr. White and red scale bars denote 20 and 10 μm, respectively. (**D**) Cells expressing the indicated constructs were subjected to *in vivo* RNA co-immunoprecipitation (RNA-coIP) using an antibody against NCL. Bound RNA was analyzed by RT-qPCR, confirming that NCL interacts only with GAr intronless RNA. Graphs represent the mean of three independent experiments. **P< 0.01, ns: not significant. (**E**) Total RNA from transfected cells was incubated with recombinant NCL *in vitro* and analyzed as in (D). NCL interacts *in vitro* with GAr-intronless and GAr-intron-bearing RNAs. Graphs represent the mean of three independent experiments. ***P< 0.001, ns: not significant.

We next set out to investigate the mechanisms whereby splicing affects GAr-mediated translation control and the interaction with NCL by evaluating mRNA localization. RNA-FISH was carried out using probes against OVA or Globin mRNAs revealing that RNAs encoded from intron-bearing or intronless RNAs without the GAr were predominantly detected in the cytoplasmic compartment, with a smaller fraction in the nucleus (Figure 2A, upper panels). Similarly, the corresponding intron-bearing GAr-carrying mRNAs were predominantly detected in the cytoplasm. On the contrary, GAr intronless messages were mainly observed in the nucleoplasm (Figure 2A, lower panels and Supplementary Figure S3). Quantification of Globin or OVA mRNAs in the cytoplasmic and nuclear fractions by RT-qPCR confirmed that the GAr-carrying intronless mRNAs are predominantly located in the nuclear compartment (Figure 2B, left and right graphs).

The GAr-encoding RNA sequence forms G4 that act as a platform for NCL binding (21). Using the proximity ligation assay (PLA) adapted to protein-mRNA interactions (34), we uncovered that NCL binds GAr-Globin intronless mRNAs in the nucleoplasm and no interactions were detected in the cytoplasm. This interaction was not observed using the GAr-Globin intron-bearing RNA, or RNAs without the GAr sequence (Figure 2C, Supplementary Figures S4 and S5). NCL binding to intronless GAr mRNAs was further confirmed by an *in vivo* RNA-CoIP assay, where NCL was immunoprecipitated (IPed) and the presence of Globin RNAs was determined using RT-qPCR (Figure 2D). Next, we tested if the GAr G4 structure was still present in the intron-bearing transcript. RNA from cells transfected with indicated constructs was subjected to an *in vitro* RNA-CoIP assay using recombinant NCL and similar interaction levels were observed between NCL and intronless or intron-bearing GAr-Globin mRNAs *in vitro* (Figure 2E). Hence, the intron-bearing constructs retain their G4 structure and the capacity to bind NCL, showing that targeting GAr*-*carrying mRNAs for splicing makes the RNA non-accessible for NCL.

### Splicing inhibition restores NCL – GAr mRNA interaction and stimulates antigenic peptide production

**Figure 3.**
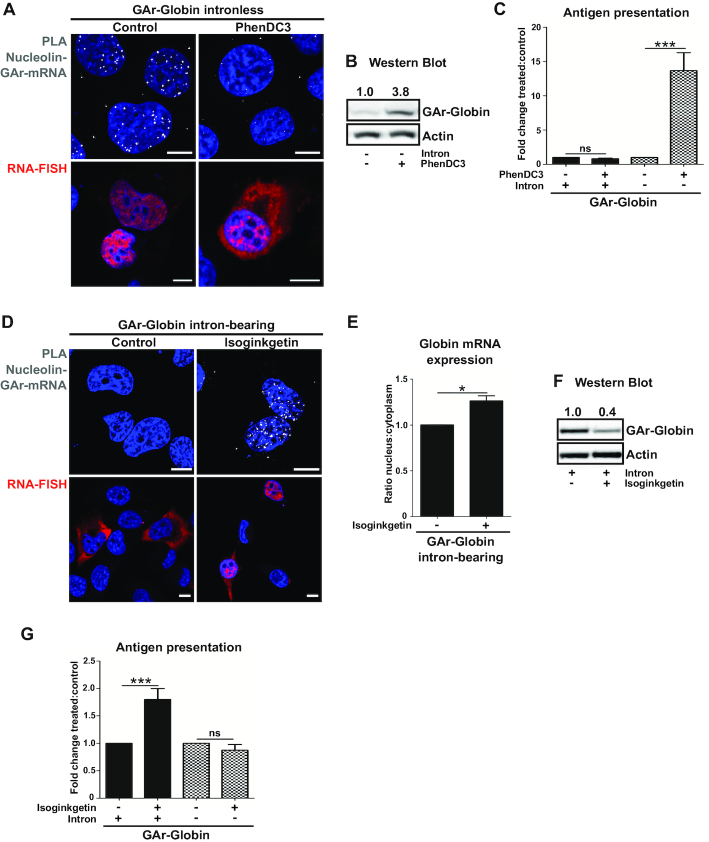
Splicing inhibition induces NCL - GAr mRNA interaction but does not affect the synthesis of antigenic peptides. (**A**) Screening of NCL - GAr mRNA interactions (white dots) by PLA (upper panels) and RNA localization by RNA-FISH (lower panels) in cells expressing intronless GAr-Globin, treated or not with PhenDC3 (5 μM for 40 h). (**B**) Western blot analysis of cells transfected and treated as in (A) show higher GAr-Globin protein levels upon PhenDC3 treatment. One of three independent experiments is shown. (**C**) H1299 cells expressing murine MHC-I (Kb) and the GAr-Globin intron-bearing or intronless constructs were treated with PhenDC3 (5 μM for 40 h) and co-cultured with OT1 CD8^+^ T cells. The levels of antigen presentation were estimated by measuring the IL-2 release. (**D**) NCL – GAr mRNA interactions by PLA (upper panels) and RNA-FISH (lower panels) in cells expressing the GAr-Globin intron-bearing mRNA, following isoginkgetin treatment (30 μM for 24 h). (**E**) Cells transfected and treated as in (D) were fractionated for analysis of RNA from the nucleus and cytoplasm by RT-qPCR. Histograms represent the mean of three independent experiments. *P< 0.05. (**F**) Western blot analysis of cells as in (D) show lower GAr-Globin protein levels upon isoginkgetin treatment. One of three independent experiments is shown. **G**) Cells were transfected and tested for antigen presentation as in (C) after treatment with isoginkgetin (30 µM for 24 h). Isoginkgetin treatment significantly increased the levels of antigen presentation from the GAr-Globin intron-bearing construct whereas no effect was observed upon PhenDC3 treatment. Blue and red represent DAPI and mRNA, respectively. Scale bar = 10 μm.

Next, we employed the NCL–GAr mRNA interaction to differentiate between synthesis of full-length proteins and antigenic peptide substrates. This interaction is prevented by the G4 ligand PhenDC3 (21). As expected, treatment of cells expressing the intronless GAr-Globin mRNAs with this compound prevented NCL binding (Figure 3A, upper panels) and increased the synthesis of both full-length protein and antigenic peptide substrates from the GAr-Globin intronless mRNA (Figure 3B and C). These effects were associated with an accumulation of GAr-intronless mRNAs in the cytoplasm (Figure 3A, lower panels). PhenDC3 had no effect on antigen presentation in cells expressing GAr-intron-bearing mRNAs, in line with the fact that these transcripts do not interact with NCL (Figure 3C).

Since targeting GAr-carrying mRNAs for splicing prevents NCL binding (see Figure 2), we next tested if splicing inhibition can restore the interaction. Treatment of cells expressing GAr-Globin intron-bearing mRNAs with the splicing inhibitor Isoginkgetin resulted in an accumulation of pre-mRNAs in the nuclear compartment and a high number of NCL – GAr mRNA interactions in the nucleus (Figure 3D, upper and lower panels, 3E and Supplementary Figure S6A). Of note, while splicing inhibition prevented the synthesis of full-length proteins (Figure 3F), it increased the synthesis of antigenic peptide substrates, despite inducing the NCL – mRNA interaction (Figure 3G). Isoginkgetin treatment had no effect on antigen presentation from the GAr-Globin intronless mRNA, confirming that the observed results are attributed to splicing inhibition and not to off-target effects of the drug (Figure 3G, Supplementary Figure S6B). These data support the idea that antigenic peptide substrates for the MHC class I pathway are produced by a mechanism independent from full-length protein synthesis. Moreover, they indicate that NCL interaction with GAr-intron-bearing RNAs upon splicing inhibition does not suppress the production of antigenic peptide substrates.

### Cytoplasmic NCL – mRNA interactions prevent canonical translation but not synthesis of antigenic peptides

**Figure 4.**
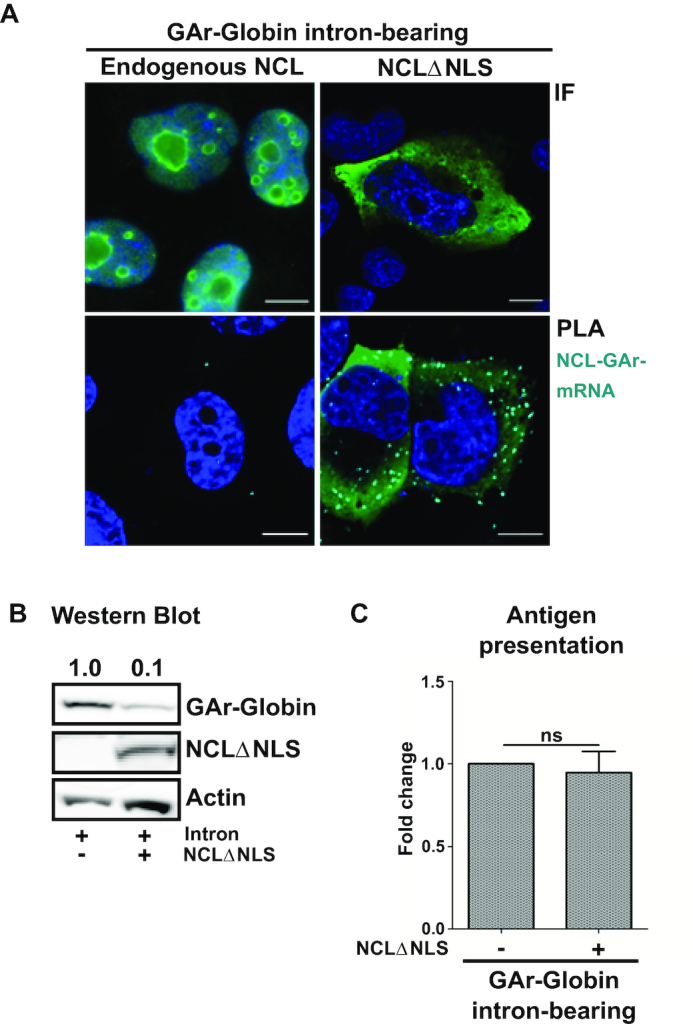
A cytoplasmic NCL suppresses synthesis of full-length protein from GAr-intron-bearing mRNAs but has no effect on antigen presentation. (**A**) Immunofluorescence (IF) shows endogenous NCL predominantly localized in the nucleolus (upper left). An anti-HA antibody shows an HA-tagged NCL lacking the nuclear localization signal (HA-NCLΔNLS) in the cytoplasm (upper right). PLA (lower panels) shows that NCLΔNLS but not endogenous NCL interacts with the GAr-Globin intron-bearing transcript in the cytoplasm. Green, dark blue and light blue represent nucleolin, DAPI and PLA, respectively. Scale bar = 10 μm. (**B**) Cells transfected as in (A) were analyzed by western blot. NCLΔNLS suppresses canonical translation of GAr-Globin intron-bearing. (**C**) Cells expressing MHC-I (Kb) and the indicated constructs were co-incubated with OT1 cells. IL-2 levels show that the expression of NCLΔNLS in the cytoplasm had no effect on the presentation of GAr-Globin intron-bearing-derived antigenic peptides. Graphs represent the mean of three independent experiments. ns: not significant.

Previous data show that PhenDC3 treatment disrupts the NCL interaction, interferes with the localization of intronless mRNAs and increases full-length protein levels and antigen presentation. Interestingly, when the nuclear retention of intron-bearing RNAs is caused by splicing inhibition, the binding of NCL does not affect the production of antigenic peptide substrates. To clarify how these different observations are inter-linked and if the effect of NCL to suppress mRNA translation is related to nuclear retention, we analyzed the effect of NCL on intron-bearing RNAs once the mRNAs had reached the cytoplasm. Spliced GAr mRNAs are typically localized in the cytoplasm (Figure 2A and B). Since NCL is found in the nucleus (Figure 4A, upper left), we created a cytoplasmic NCL construct by disrupting the nuclear localization sequence of an HA-tagged NCL (HA-NCLΔNLS) (Figure 4A, upper right). When we performed PLA in cells expressing HA-NCLΔNLS together with GAr-Globin intron-bearing RNAs, we observed the HA-NCLΔNLS – GAr mRNA interaction in the cytoplasm (Figure 4A, lower right). As expected, the endogenous NCL did not interact with the intron-bearing GAr-Globin mRNA (Figure 4A, lower left).

The presence of HA-NCLΔNLS hampered the synthesis of full-length proteins from the GAr intron-bearing mRNAs (Figure 4B), but it had no effect on the production of antigenic peptide substrates (Figure 4C). Hence, when NCL interacts with the GAr mRNA in the cytoplasm it only interferes with the canonical translation and not with the production of antigenic peptide substrates. These results support the notion that translation events taking place in the cytoplasm do not affect the production of antigenic peptide substrates for the MHC class I pathway.

### Altering the 5′ UTR of GAr-carrying mRNAs affects their ability to interact with NCL

**Figure 5.**
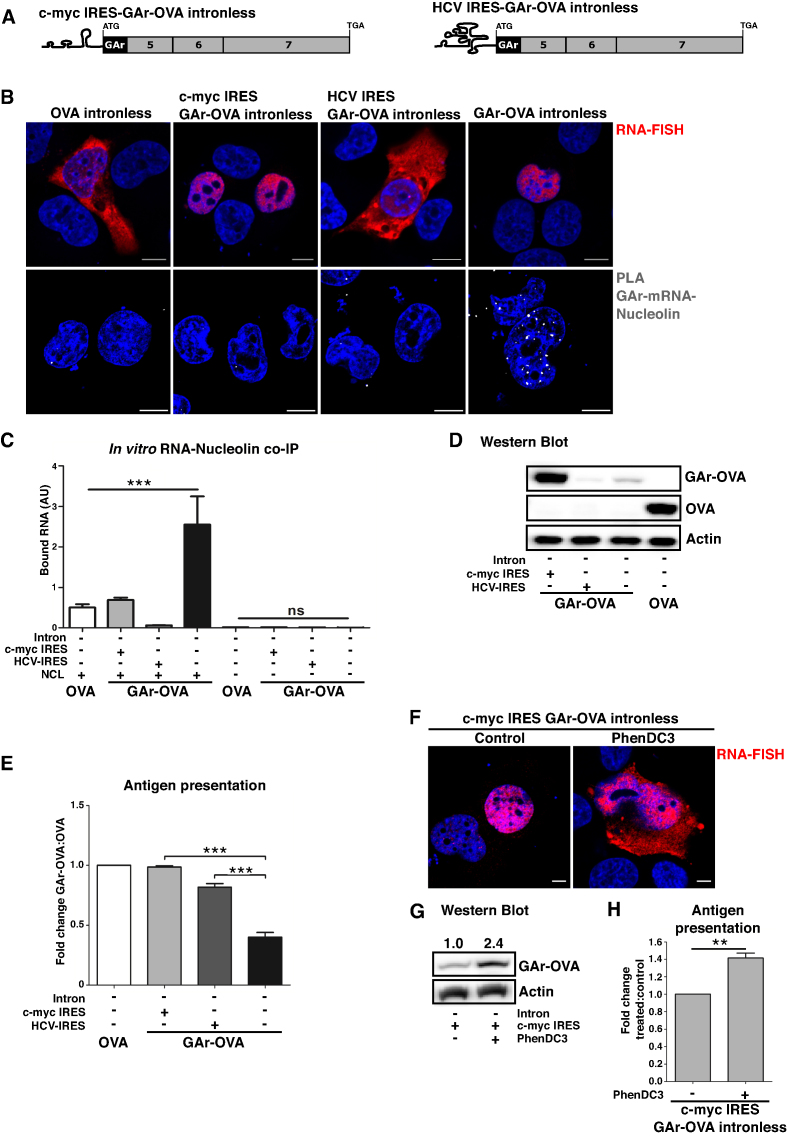
IRES sequences fused to the 5′ UTR of GAr-intronless mRNAs disrupt G4 function and differentiate synthesis of full-length vs antigenic peptide products. (**A**) Cartoon illustrating c-my*c* IRES-GAr-OVA intronless and HC*V* IRES-GAr-OVA intronless constructs. (**B**) RNA FISH (upper panels) and PLA analysis (lower panels) of NCL – GAr mRNA interactions (depicted as white dots) in cells expressing the indicated constructs. c-myc IRES GAr-OVA and HCV IRES GAr-OVA intronless mRNAs show different locations (upper panels). The presence of either IRES sequences prevents the interaction with NCL (lower panels). (**C**) *In vitro* RNA co-immunoprecipitation (RNA-coIP) on total RNA derived from cells expressing the indicated constructs. Graphs represent the mean of three independent experiments. ***P< 0.001, ns: not significant. (**D**) Western blots show the protein levels from intronless OVA, GAr-OVA and IRES-carrying GAr-OVA constructs (see also Supplementary Figure S7). (**E**) Antigen presentation determined by IL-2 release from OT-1 cells co-incubated with cells expressing murine MHC-I (Kb) and indicated constructs. Graphs represent the mean of three independent experiments. ***P< 0.001. (**F**) RNA-FISH on cells expressing intronless c-myc IRES GAr-OVA treated with PhenDC3 (5 μM for 40 h). (**G**) Western blot on cells transfected and treated as in (F). One of three independent experiments is shown. (**H**) Cells expressing murine MHC-I (Kb) and the c-myc IRES GAr-OVA intronless construct were treated with PhenDC3 and tested for antigen presentation as in (E). Blue represents DAPI. Scale bar = 10 μm.

Previous reports have shown that fusing the c-myc IRES to the 5′ UTR of intronless GAr-carrying mRNAs overcomes GAr-mediated translation suppression (2). We added c-myc and HCV IRES structures to the 5′ UTR of GAr-carrying messages (Figure 5A) and RNA-FISH analysis showed that the c-myc IRES had no effect on the localization of the intronless GAr mRNA whereas the HCV IRES stimulated nuclear export (Figure 5B, upper panels). Screening of NCL – GAr mRNA interactions using the PLA showed that both IRESs prevented the binding of NCL (Figure 5B, lower panels). In addition, *in vitro* RNA co-IP using total RNA from transfected cells and recombinant NCL showed that neither the c-myc nor the HCV IRES-carrying mRNAs interacted with NCL (Figure 5C). This shows that IRES sequences in the 5′ UTR of the GAr-carrying mRNAs prevent their interaction with NCL both *in vitro* and *in cellulo*, presumably by interfering with the formation of the G4 structure.

As expected, the c-myc IRES overcame GAr-mediated translation suppression and had no impact on translation efficiency of non GAr-carrying mRNAs. The presence of the HCV IRES, however, further suppressed protein synthesis, which can be explained by the fact that the HCV IRES forms a hairpin structure that prevents cap-dependent translation also in the absence of the GAr (Figure 5D and Supplementary Figure S7). Importantly, both IRESs prevented GAr-mediated suppression of antigen presentation (Figure 5E). Hence, the HCV IRES stimulates antigen presentation while suppressing canonical translation, further supporting the notion that full-length proteins and antigenic peptide substrates are derived from distinguishable translation events.

We have previously shown that NCL suppresses translation when bound to the G4 structures present in GAr-encoding mRNA, but we also observed that treating cells with PhenDC3 induced the export of GAr-intronless transcripts to the cytoplasm (see Figure 3A and B). This raised the possibility that NCL might anchor the mRNA in the nucleus. To test this, we treated cells expressing the c-my*c* IRES-GAr mRNA with PhenDC3 and analyzed RNA location by FISH. In non-treated cells this mRNA remained nuclear in spite of not interacting with NCL, but following PhenDC3 treatment it was predominantly detected in the cytoplasm (Figure 5F). This shows that other G4-binding factor/s prevent mRNA export. Interestingly, the nuclear export of c-myc IRES-GAr mRNA promoted by PhenDC3 was associated with an increased synthesis of both protein and antigenic peptide substrates (Figure 5G and H). These data show that the GAr G4 can act as a hub for different cellular factors that interfere with mRNA processing in varied ways.

### Altered mRNA export pathway prevents the interaction between NCL and intronless GAr-carrying mRNAs

**Figure 6.**
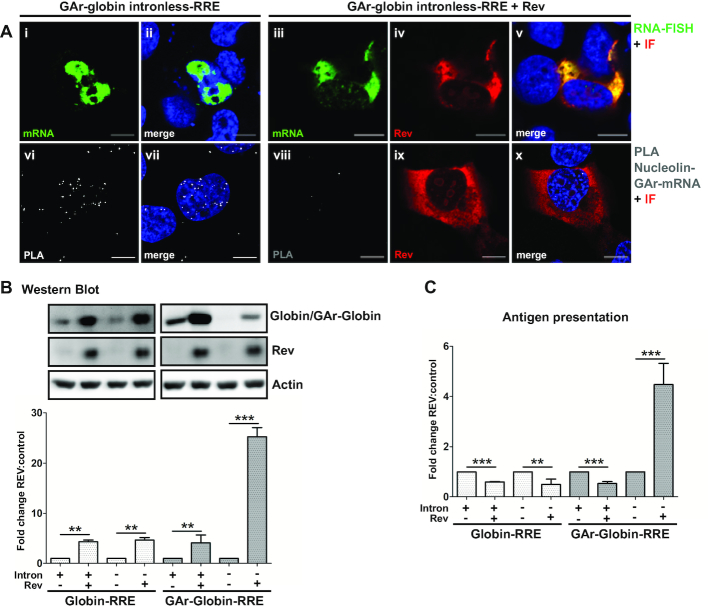
Rev-mediated nuclear-export prevents NCL - GAr-intronless mRNA interaction. (**A**) RNA FISH coupled to IF (upper panels) and PLA analysis of NCL – GAr mRNA interactions coupled to IF (lower panels) in cells expressing indicated constructs. Upper panels: The Rev response element (RRE) sequence was fused to the 3′UTR of GAr-Globin intronless (GAr-Globin intronless-RRE) (see also Supplementary Figure S8A). The GAr-Globin intronless-RRE mRNA is exported to the cytoplasm in a Rev-dependent fashion (iii–v). Lower panels: The GAr intronless mRNA does not interact with NCL following nuclear export by Rev (viii–x). PLA complexes are depicted as white dots and each complex represent an interaction between NCL and GAr-mRNA. Green and red denotes GAr mRNA and Rev, respectively. Blue represents DAPI. Scale bar = 10 μm. (**B**) Western blot analysis (upper panels) of cells expressing Rev or empty vector and indicated intronless or intron-bearing constructs. Nuclear export of mRNAs by Rev results in higher protein expression. Graph (lower) shows fold changes of Globin:actin protein levels relative to controls without Rev and represent three independent experiments. **P< 0.01, ***P< 0.001. (**C**) Antigen presentation levels were determined by measuring the IL-2 release by OT-1 lymphocytes co-incubated with cells expressing murine MHC-I (Kb) and the same constructs as in (B). Nuclear export of mRNAs by Rev suppresses the synthesis of antigenic peptide substrates except for the intronless GAr-Globin-RRE construct, which shows an ∼5-fold increase of antigen presentation. Data shown are fold changes relative to controls without Rev and represent three independent experiments. **P< 0.01, ***P< 0.001.

Presented results suggest that distinct maturation mechanisms that restrain mRNAs in the nucleus, or drive them to cytoplasm, control the synthesis of full-length protein and antigenic peptide substrates. To test the role of mRNA export pathways, we targeted the nuclear intronless GAr mRNA for the CRM1 export pathway using the HIV-1 RRE-Rev system. RNA-FISH revealed that the insertion of the RRE sequence to the 3′ UTR of GAr-Globin intronless mRNA (GAr-Globin intronless-RRE) did not result in any change in its subcellular localization (Figure 6A, i–ii and see Figure 2A). The RRE itself had no effect on the NCL – GAr mRNA interaction (Figure 6A, vi–vii). In the presence of the Rev protein, intronless GAr-Globin-RRE mRNA was exported to the cytoplasm (Figure 6A, iii-v) and the interaction with NCL was lost (Figure 6A, viii–x). The presence of Rev also enhanced the rate of protein expression from GAr-carrying and non-GAr-carrying RRE mRNAs, with a nearly 25-fold induction of expression from the GAr-Globin intronless-RRE (Figure 6B, upper panels and lower graph). When we looked at the effect of Rev on the production of antigenic peptide substrates, we observed a significant suppression from all mRNAs tested with the clear exception of the intronless GAr-Globin-RRE, for which an ∼5-fold induction was uncovered (Figure 6C). Thus, Rev-mediated export of mRNAs originally located in the cytoplasm improved their rate of translation but at the same time reduced the synthesis of antigenic peptides. Importantly, the forced export of the nuclear-retained intronless GAr-carrying mRNAs prevented NCL binding and promoted a concurrent increase of both canonical translation and antigen presentation. These results support the hypothesis that protein-RNA intereaction taking place on the nascent transcript control the synthesis of full length proteins and antigenic peptide substrates.

## DISCUSSION

We have employed multiple approaches to show that the synthesis of full-length proteins and antigenic peptide substrates for the MHC class I pathway are separate events independently regulated on the nascent transcript.

**Figure 7.**
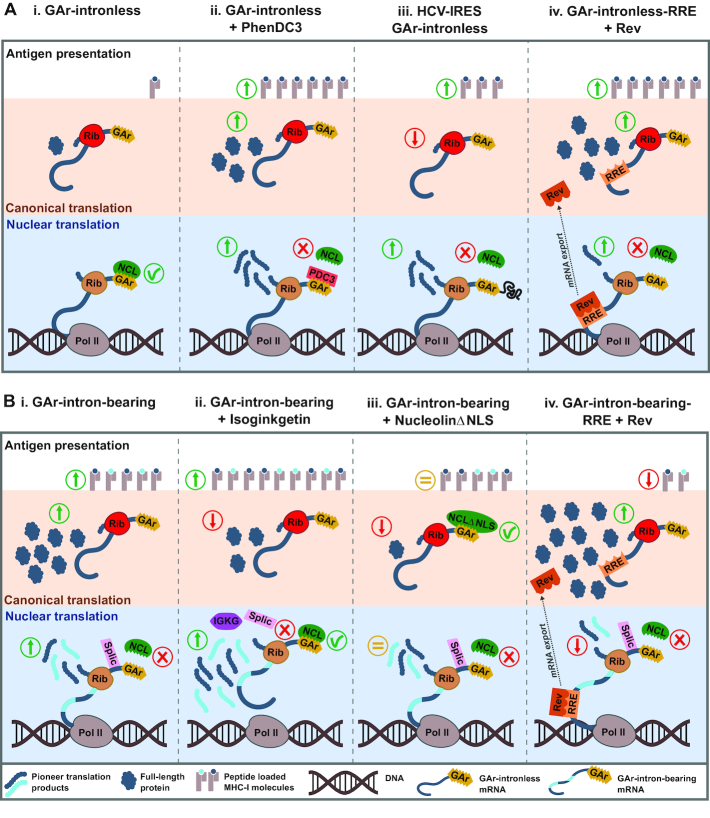
Scheme of the effects of G4 ligands, splicing and alternative mRNA export on the synthesis of full-length proteins and pioneer translation products (PTPs) from intronless and intron-bearing GAr-carrying transcripts. (**A**) GAr-intronless mRNAs. (A.i) GAr-mediated inhibition of full-length proteins and PTPs production depend on the binding of Nucleolin (NCL) to G-quadruplexes (G4) formed by GAr-encoding mRNA in the nucleus. (A.ii) The G4-ligand PhenDC3 prevents the NCL - GAr-mRNA interaction, promoting increased protein and antigen presentation levels. (A.iii) Fusion of the HCV IRES structure to the 5′UTR of GAr-intronless mRNAs hampers NCL - GAr-mRNA interactions resulting in an increase of PTPs production concurrent with reduced full-length protein levels. (A.iv) HIV-1 Rev-mediated mRNA export prevents NCL binding to the GAr mRNA resulting in increased full-length protein and PTPs production. In (A.ii) to (A.iv), changes are illustrated taking (A.i) as reference. (**B**) GAr-intron-bearing mRNAs. (B.i) NCL does not interact with GAr-carrying mRNAs targeted by the splicing machinery (Splic). GAr-mediated control of mRNA translation and antigen presentation is disrupted in the context of intron-bearing messages (changes illustrated taking A.i as reference). (B.ii) Splicing inhibition by Isoginkgetin (IGKG) reduces protein levels but promotes an increase in PTPs production. IGKG prevents mRNA maturation allowing non-spliced pre-mRNAs to interact with NCL after the production of PTPs has taken place. (B.iii) A NCL defective for nuclear localization (NCLΔNLS) interacts with spliced GAr-mRNAs in the cytoplasm, resulting in a strong decrease in full-length protein synthesis without affecting PTPs production. (B.iv) Rev-mediated export of GAr-intron-bearing mRNA prevents the production of PTPs in the nucleus and reduces antigen presentation levels in spite of increasing canonical translation. In B.ii to B.iv, changes are illustrated taking B.i as reference. Green and red arrows denote increased and decreased levels, respectively. Yellow equals signs indicate no significant change. Ribosomes (Rib) translating PTPs and full-length proteins are depicted in brown and red, respectively. Dark and light blue lines denote exonic and intronic RNA sequences, respectively. Pol II: RNA polymerase II.

Translation in the nuclear compartment is still clouded in controversy despite several reports suggesting that it indeed can take place (35–42). In support of a nuclear non-canonical translation event for the production of antigenic peptide substrates, we show that (i) splicing inhibition promotes an increase of antigen presentation in spite of reducing protein levels, (ii) the interaction between NCL and mRNAs in the cytoplasm prevents synthesis of full-length proteins but has no effect on the production of antigenic peptide substrates, (iii) the HCV IRES overcomes GAr-mediated inhibition of antigenic peptides production while suppressing the synthesis of full-length proteins and iv) alternative mRNA export via the CRM1-dependent pathway of non-GAr-carrying intronless messages stimulates protein synthesis but suppresses antigen presentation (Figure 7). These results are in line with previous reports showing that mRNAs targeted for the NMD pathway produce antigenic peptides substrates but not full-length proteins and that nascent intron-derived peptides are detected in the nucleus and presented on MHC class I molecules (8). The synthesis of antigenic peptide substrates might relate to previous published observations of ribosomal factors on nascent mRNAs and of coupled transcription-translation in eukaryotic cells (37,41,42). Furthermore, mass spectrometry analyses have also shown that a large proportion of small peptide products are derived from non-canonical translation (13). A non-canonical translation taking place in the nucleus might not require the same translation factors as the synthesis of full-length proteins, which takes the edge off some of the arguments against nuclear translation. Taken together, previous and presented data suggest a model in which a major source of antigenic peptide substrates for the MHC class I pathway are synthesized by a nuclear event taking place on pre-spliced mRNAs (7,8,43). However, this does not rule out other sources of peptide substrates for the MHC class I pathway (44).

Pre-mRNAs are processed and exported to the cytoplasm as translation competent mRNAs (14,45). Nuclear mRNA retention is considered rare in mammalian cells (46) and few RNA sequences promoting nuclear retention have been identified (47). Here, we show that in addition to provide a NCL-binding platform, the G4 structure of the GAr-encoding mRNA also promotes nuclear retention. This supports previous findings that the G4 acts as a protein binding platform (21) and not as an inhibitor of translation elongation (48). The relevance of RNA G4 in the physiological context has been a matter of debate. However, the observation that these structures are thermodynamically very stable under near physiological ionic conditions *in vitro* strongly suggests that they are likely to form *in vivo* (25) and accumulating evidence from *in cellulo* experiments demonstrate that RNA G4 play a relevant role in the gene regulation process (20,21,25,48). In accordance with our observations, Serikawa *et al.* (20) showed that NCL binds RNA G4 motifs found in tumor-associated mRNAs *in cellulo*. Moreover, putative quadruplex sequences have been identified in the mRNAs coding other gammaherpesviral maintenance proteins similar to EBNA1 (48). Therefore, it is tempting to infer that NCL - G4 interactions play a relevant role in tumor biology and might be exploited by viruses to control mRNA translation and antigen presentation.

Both capacities of the GAr G4 to interact with NCL and promote mRNA nuclear retention are disrupted by the G4 ligand PhenDC3, but these two functions can also be targeted independently by fusing the c-myc or the HCV IRESs to the 5′ UTR of GAr-carrying mRNAs. Both IRESs disrupt the capacity of the G4 structure to bind NCL *in vitro* and *in vivo* but the c-myc IRES does not interfere with nuclear retention, whereas HCV IRES-carrying mRNAs are exported freely. This indicates that the nuclear retention factor has a different binding affinity for the G4 as compared to NCL, supporting the idea that the folding of G4 RNA structures *in vivo* can take different conformations and is influenced by flanking sequences (49,50). Lago *et al.* (17) observed that NCL modifies the overall structure and steric hindrance of G4 forming sequences. Therefore, modifications of the GAr G4 structure induced by NCL, or factor/s promoting nuclear retention, could represent a mechanosensor governing mRNA maturation. Interestingly, the intron-bearing G4 RNA binds a cytoplasmic NCL (NCLΔNLS) and suppress translation, despite the fact that the mRNA has been scanned during RNA quality control, showing that the G4 structure is refolded after the pioneer round of translation. The RNA helicase RHAU has been shown to bind mRNAs *in cellulo*, unwind G4 structures and function as the main source of tetramolecular RNA G4-resolving activity in HeLa cells lysates (51,52). A similar activity has been described for the human DHX9 helicase (53).

The interaction with NCL was also prevented by placing the GAr in the context of intron-bearing mRNAs. However, in this case the structure of the G4 is not disrupted since GAr*-*intron bearing transcripts interact with NCL *in vitro* and splicing inhibition allows NCL to access the G4 in the nucleus. Therefore, engaging the mRNA with the splicing machinery prevents NCL access to the nascent transcript. Upon splicing inhibition, NCL binding to intron-containing RNAs in the nucleus does not affect the production of antigenic peptides. This indicates that NCL must interact with nascent transcripts in order to suppress the production of antigenic peptides and reinforces the idea that PTPs are produced co-transcriptionally (8).

As we observed that G4-carrying intron-bearing RNAs are freely exported, it is possible that splicing also prevents the access of a nuclear retention factor/s to G4. It should be noted that EBNA1 has been reported to bind G4-forming RNAs in order to recruit the origin recognition complex (ORC) and modulate EBV DNA replication and episome maintenance (54). Therefore, one cannot exclude the possibility that EBNA1 interferes with translation and antigen presentation by binding G4 structures in its own mRNA.

There is yet little known about alternative and regulated mRNA maturation pathways. However, these results indicate that the formation of specific RNP complexes on the nascent transcripts control mRNA maturation pathways that determine how the messages will be translated in the cytoplasm. In line with this notion, NCL is under normal conditions only bound to GAr-carrying mRNAs in the nucleus but it still prevents canonical translation in the cytoplasm. This notion is also illustrated by Rev-mediated mRNA export via the CRM1 pathway (Supplementary Figure S8A and B). Rev targets pre-spliced mRNAs for nuclear export and this prevents NCL from binding the GAr G4 structure, resulting in an increase in the antigenic peptides production. However, Rev-mediated nuclear export of non-GAr-carrying mRNAs instead results in a sharp loss of antigenic peptides production. This might appear contradictory, but one has to keep in mind that the actual numbers of antigenic peptides derived from GAr intronless mRNAs following Rev-mediated export are far less than the number of peptides derived from non-GAr-carrying messages. Hence, Rev-mediated export of GAr-carrying mRNAs does not restore antigenic peptide production: it merely increases the numbers from very low to low. In this scenario, a Rev-dependent loss of NCL binding is sufficient to allow the production of PTPs before the message is exported. Mature mRNAs are also efficiently exported by Rev, leading to higher levels of full-length proteins. It cannot be ruled out that the minor increase of antigen presentation from GAr fusion proteins observed from Rev-dependent export might be associated to a protein-based source of peptides.

It was surprising to see that the c-myc IRES-carrying mRNAs overcome GAr-mediated suppression of full-length protein synthesis while retaining a mainly nuclear localization. It might have been expected that an increase in synthesis would have been accompanied with nuclear export. Thus, nuclear retention *per se* is not sufficient to inhibit translation and this presumable relates to previous observations that a fraction of mRNAs are engaged with translation. Taken together, we suggest a model whereby GAr is targeting NCL to intronless mRNAs and this suppresses the synthesis of PTPs in the nucleus as well as full-length proteins in the cytoplasm (Figure 7). It is possible that the function of the GAr is to promote an mRNA maturation pathway which allows G4 to engage with NCL and suppress mRNA translation. Additionally, this alternative pathway might enable the binding of other factor/s to the G4 structure, leading to nuclear retention. Further studies will clarify the implication of nuclear retention in the viral strategy to evade the immune system.

NCL is a multifunctional RNA binding protein that suppresses translation of multiple mRNAs (16,24,55). Nevertheless, the underlying molecular mechanism of this inhibitory effect is poorly understood. The presented model for how the GAr governs translation during early mRNA maturation shows some similarities with the alternative mRNA nuclear export (ALREX) concept. ALREX-elements located in the 5′ of mRNAs coding sequences control translation, transcript export and encode a signal sequence, which guides proteins to the endoplasmic reticulum (ER) (56). EBNA1 or the GAr-carrying proteins that we have used are not targeted to the ER. Nevertheless, the fact that the GAr mRNA sequence controls mRNA export and translation suggests a more general concept in which mRNA maturation, export and translation are interlinked. In the case of EBNA1, the interaction between the RNA and nucleolin in the nucleoplasm controls both the production of antigenic peptide substrates and canonical cytoplasmic translation. For the RanBP2/Nup358, the interaction with ALREX-elements takes place at the nuclear membrane. In either case, NCL and RanBP2/Nup358 are likely to affect translation by modifying mRNP complexes that govern translation initiation before the mRNAs reach the cytoplasm. Their respective effects on canonical translation presumably take place before they are displaced by the scanning ribosomes during the first round of pioneer translation.

The design of new vaccines against transformed or virus-infected cells relies on the presentation of neoantigens on MHC class I molecules. Thus, the presented results will lead to a better understanding of the underlying molecular mechanisms that control the synthesis of peptide substrates for the MHC class I pathway and pave the way for the development of new therapeutic approaches aimed at regulating the presentation of antigens in pathological conditions.

## Supplementary Material

Supplementary Data
