# Supplementary material for: Nuclear processing of nascent transcripts determines synthesis of full-length proteins and antigenic peptides
Source: Nucleic Acids Res. 2019 Jan 9;47(6):3086–100. doi: 10.1093/nar/gky1296 (PMC6451098; doi:10.1093/nar/gky1296)
Supplement: Supplementary Data [file gky1296_supplemental_files.pdf]

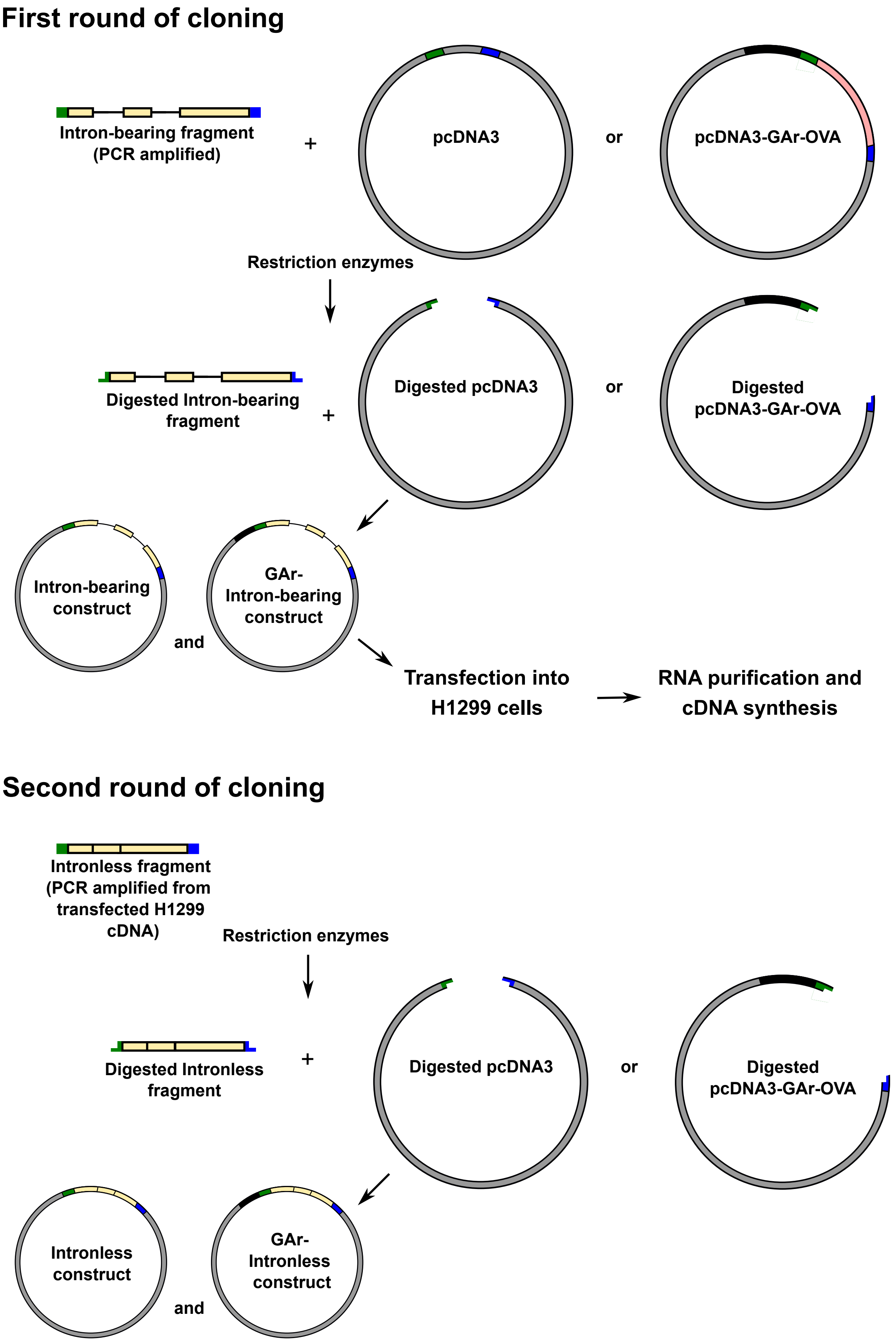

Supplementary Figure 1 – Cloning approach employed for producing the splicing reporters used in this study. In the first round of cloning, full-length globin intron-bearing and OVA exon 5-7 fragment were PCR-amplified from the Glob-exon-SL8 construct (Apcher et al. 2013) and chicken genomic DNA, respectively. The amplicons were cloned into pcDNA3 to create the control constructs or replaced the OVA ORF of the pcDNA3-GAr-OVA construct (Yin et al. 2003) to generate plasmids with the GAr domain upstream and in the same frame of the inserts. In the second round of cloning, the intronless fragments were PCR amplified using the same primers employed in the previous round of cloning and cDNAs obtained from H1299 cells transfected with the intron-bearing constructs. Afterwards, pcDNA3 or pcDNA3-GAr-OVA construct were employed as vectors to produce intronless or GAr-intronless constructs. Green and blue rectangles denote 5'and 3' restriction sites. Black and pink lines represent GAr and OVA ORFs, respectively. This relates to Figure 1.

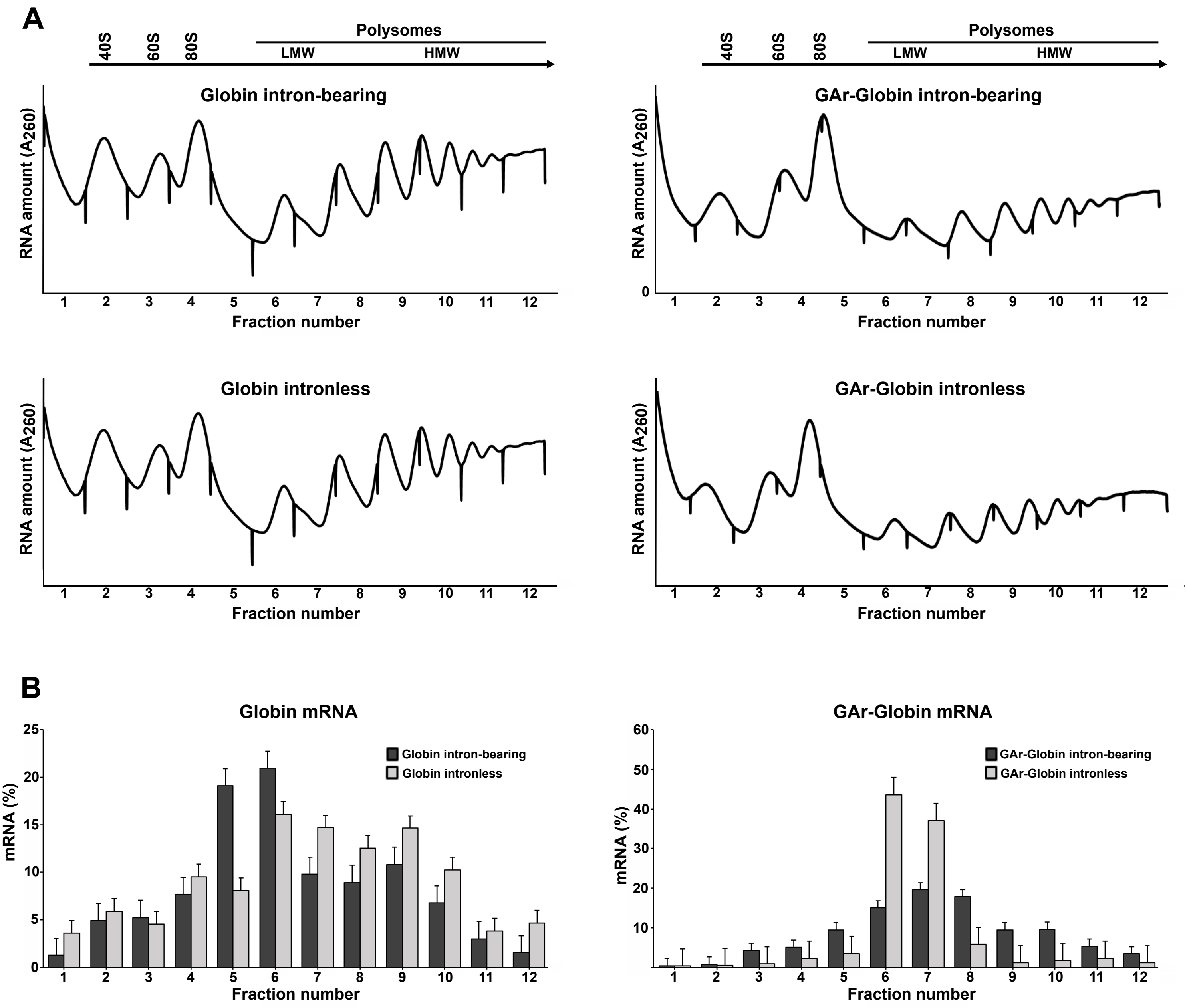

Supplementary Figure 2 – (A) Cytoplasmic lysates from cells transiently expressing indicated constructs were fractionated through sucrose gradients. Global RNA polysome profiles generated by density gradient fractionation are shown. (B) RT-qPCR analysis of indicated mRNAs in 12 collected gradient fractions. The relative distribution of target mRNAs is shown as % and was calculated using fraction 1 as reference. Left panel shows that in the absence of the GAr, intronless mRNAs are more prevalent in heavy polysomes as compared to intron-bearing counterparts. This supports data in Figures 1C, 1D and 1E suggesting that intronless mRNAs are more efficiently translated. On the other hand, the right panel shows that GAr-fused intronless mRNAs accumulate in light polysomes and are poorly detected in heavy polysomes. This reinforces previous data showing that the GAr interferes *in cis* with translation initiation. Cells expressing Globin and GAr-globin constructs were analysed in independent assays. Data relates to Figure 1.

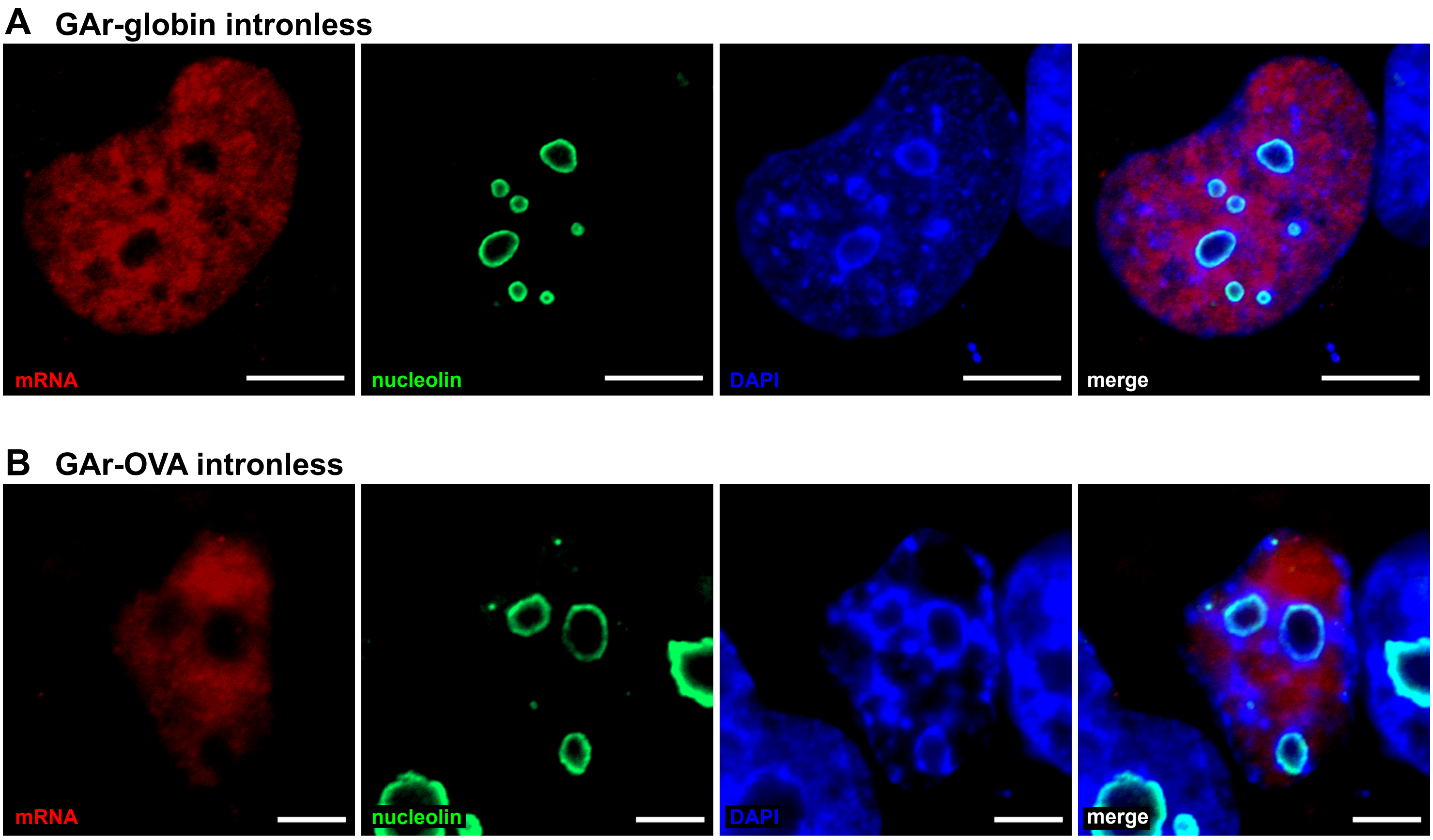

Supplementary Figure 3 – Cells expressing indicated constructs were analyzed by RNA-FISH coupled to nucleolar staining using probes against Globin (A) or OVA (B) mRNAs. GAr-carrying intronless mRNAs are observed in the nucleoplasm but not in nucleoli. Red, green and blue represent mRNAs, nuclear (DAPI) and nucleolar (nucleolin-A488) staining, respectively. Scale bar = 10 µm. Data relates to Figure 2.

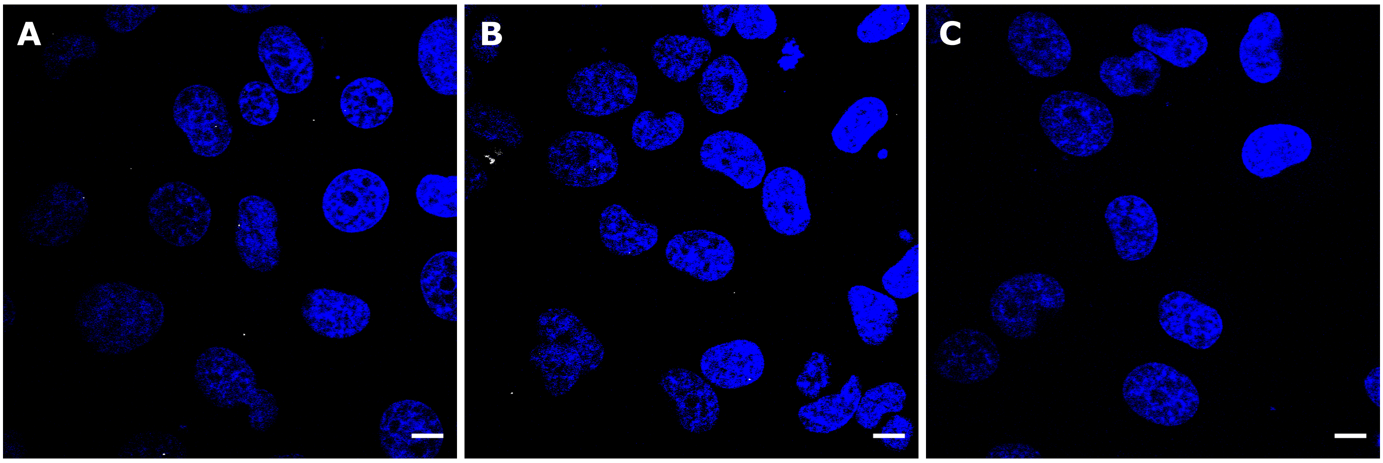

Supplementary Figure 4 – Controls of Proximity ligation assay (PLA) for screening nucleolin-GAr mRNA interactions. Non-transfected cells (A). GAr-globin intronless transfected cells tested in absence of probe targeting globin RNA (B) or anti-nucleolin antibody (C). Blue denotes DAPI. Scale bar = 10 μm. Data relates to Figure 2.

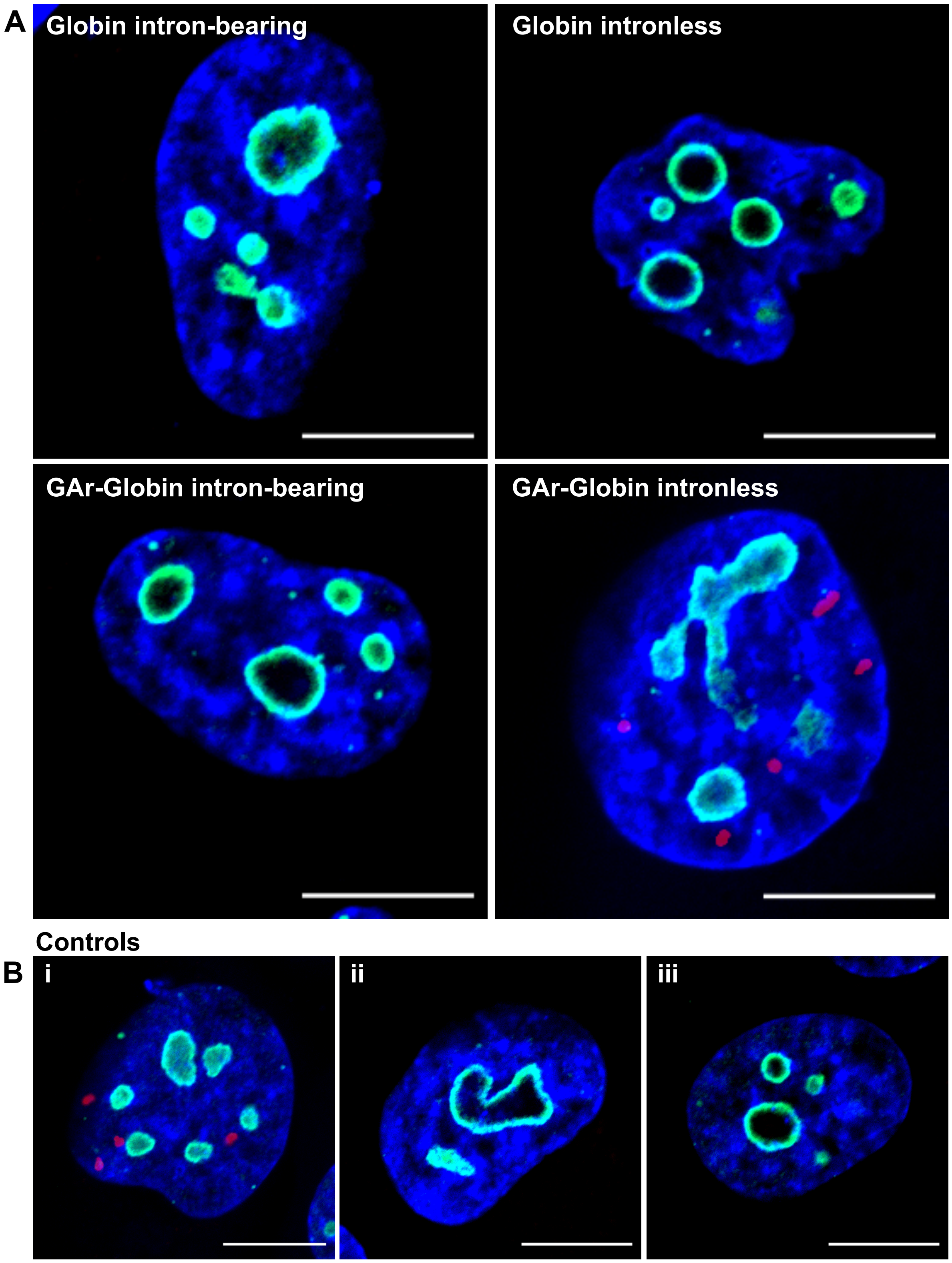

Supplementary Figure 5 – (A) Proximity Ligation Assay (PLA) on fixed cells expressing indicated constructs using anti-nucleolin (NCL) and digoxigenin-tagged globin RNA probes. Data show a nucleoplasm interaction (red dots) between nucleolin and GAr-carrying intronless mRNAs (bottom right). Blue represents nuclear (DAPI) and green nucleolar (fibrillarin-A488) staining. (B) Lower panels show PLA controls. (i): GAr-globin intronless transfected cells with anti-nucleolin and globin RNA probes. (ii): as in (i) without globin RNA probes. (iii): as in (i) without anti-NCL antibody. Scale bars 10 µm. Data relates to Figure 2.

A

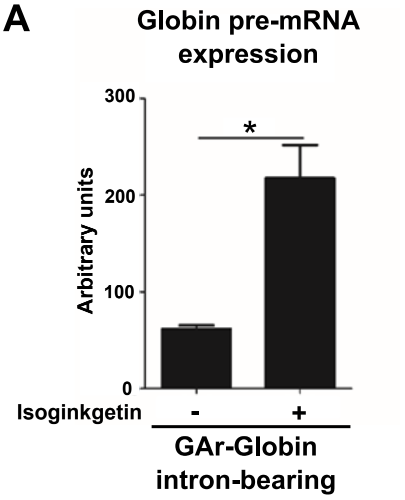

B

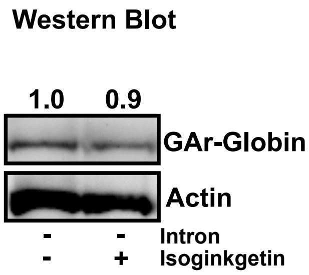

Supplementary Figure 6 – (A) Mock or Isoginkgetin-treated cells transiently expressing GAr-globin intron-bearing were analyzed by RT-qPCR using random hexamers and primers targeting globin intron 1. An accumulation of globin pre-mRNA was observed upon Isoginkgetin-mediated splicing inhibition. Histograms represent the mean of three independent experiments. *p<0.05. (B) Mock or Isoginkgetin-treated cells transiently expressing GAr-globin intronless were analyzed by western blot. Isoginkgetin treatment did not alter GAr-globin intronless protein levels. One of three independent experiments is shown. Data relates to Figure 3.

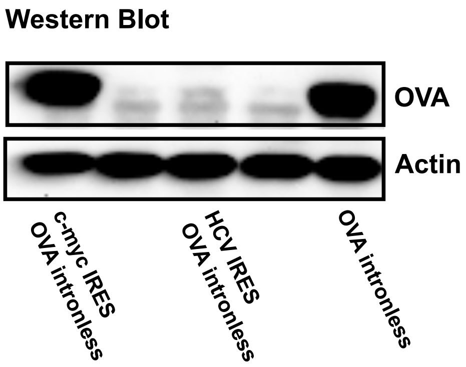

Supplementary Figure 7 – Protein levels of OVA intronless with and without the fusion of c-myc and HCV IRESs to its 5' UTR. Data relates to Figure 5.

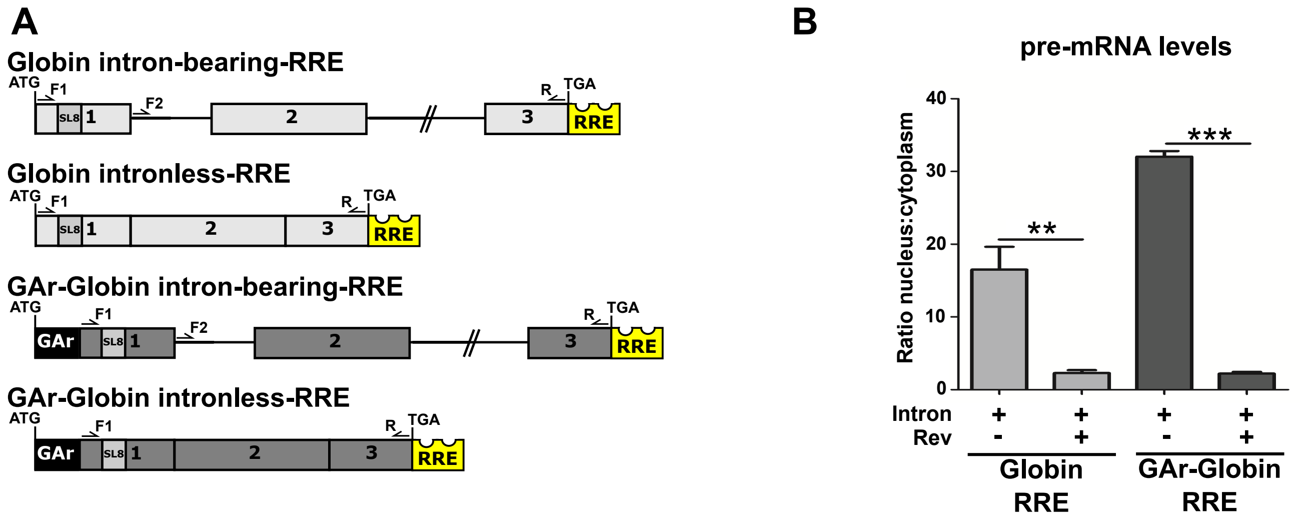

Supplementary Figure 8 – (A) Cartoons depicting RRE reporter constructs. (B) Cells expressing Rev or pcDNA3 and the indicated constructs were fractionated for the analysis of cytoplasmic and nuclear mRNA by RT-qPCR. Nuclear export mediated by Rev results in the transport of non-spliced pre-mRNA to the cytoplasm. Data relates to Figure 6.

Supplementary Table 1 – DNA constructs, primers and restriction enzymes used in this study

**Constructs**	**Primers (5'→3')**	**Restriction enzymes**	**Reference**
Globin intron-bearing	TTGAATTCGTGATGGTGCACCTGACTCCTGA<br>TAGGGCCCTTACTGGAAGGCAGCCTG	EcoRI / ApaI	This study
Globin intronless	TTGAATTCGTGATGGTGCACCTGACTCCTGA<br>TAGGGCCCTTACTGGAAGGCAGCCTG	EcoRI / ApaI	This study
GAr-globin intron-bearing	GCGAATTCGTGCACCTGACTCCTGA<br>TAGGGCCCTTACTGGAAGGCAGCCTG	EcoRI / ApaI	This study
GAr-globin intronless	GCGAATTCGTGCACCTGACTCCTGA<br>TAGGGCCCTTACTGGAAGGCAGCCTG	EcoRI / ApaI	This study
Globin intron-bearing-RRE	……	……	Apcher et al. (2013)
Globin intronless-RRE	TTGAATTCGTGATGGTGCACCTGACTCCTGA<br>TTGGGCCCTCTAGATACTCCAACTAGCA	EcoRI / ApaI	This study
GAr-globin intron-bearing RRE	GCGAATTCGTGCACCTGACTCCTGA<br>TTGGGCCCTCTAGATACTCCAACTAGCA	EcoRI / ApaI	This study
GAr-globin intronless-RRE	GCGAATTCGTGCACCTGACTCCTGA<br>TTGGGCCCTCTAGATACTCCAACTAGCA	EcoRI / ApaI	This study
OVA intron-bearing	GCGCCTCGAGATGGGAATTATCAGAAATGTCCTTCAGCC<br>TTAGGGCCCTTAAGGGGAAACACATCTGCC	XhoI / ApaI	This study
OVA intronless	GCGCCTCGAGATGGGAATTATCAGAAATGTCCTTCAGCC<br>TTAGGGCCCTTAAGGGGAAACACATCTGCC	XhoI / ApaI	This study
GAr-OVA intron-bearing	GCGCCTCGAGGGAATTATCAGAAATGTCCTTCAGCC<br>TTAGGGCCCTTAAGGGGAAACACATCTGCC	XhoI / ApaI	This study
GAr-OVA intronless	GCGCCTCGAGGGAATTATCAGAAATGTCCTTCAGCC<br>TTAGGGCCCTTAAGGGGAAACACATCTGCC	XhoI / ApaI	This study
c-myc IRES OVA intronless	……	……	Apcher et al. (2010)
HCV IRES OVA intronless	GCGCGGATCCTCCCCTGTGAGGAACTACTGT<br>TACGGGATCCGATGCACGGTCTACGAGACCT	BamHI/BamHI	This study
HA-NCLΔNLS	AAACAGAAAGCAGCTCCTGAAGCCGCGGCACAGGCAGTGGAAGGCACAGAACCGACTAC<br>GTAGTCGGTTCTGTGCCTTCCACTGCCTGTGCCGCGGCTTCAGGAGCTGCTTTCTGTTT	……	This study
REV	……	……	Gift from Ali Saïb, Saint-Louis Hospital, Paris, France.
Kb	……	……	Gift from C. Watts, University of Dundee, Dundee, UK.

Supplementary Table 2 – Primers used for RT-PCR and RT-qPCR

Primer	Sequence (5'-3')
Figure 1B	
Globin F1	ATGGTGCACCTGACTCCTGA
Globin F2	TATCAAGGTTACAAGACAGG
Globin R	CTGGAAGGCAGCCTG
OVA F1	GGAATTATCAGAAATGTCCTTCAGCC
OVA F2	GGGCATACCTTAGAGATGTAATCTAG
OVA R	TTAAGGGGAAACACATCTGCC
Figures 1C and 2B	
OVA exon 6 FW	GCAAACCTGTGCAGATGATG
OVA exon 6 RV	CTGCTCAAGGCCTGAGACTT
Globin exon 2 FW	CTGCTGGTGGTCTACCCTTG
Globin exon 2 RV	AGCTTGTCACAGTGCAGCTC
Figures 2D, 2E and 3B<br>Suppl Figure 2B	
Globin exon 2 FW	CTGCTGGTGGTCTACCCTTG
Globin exon 2 RV	AGCTTGTCACAGTGCAGCTC
Figure 5E	
OVA exon 6 FW	GCAAACCTGTGCAGATGATG
OVA exon 6 RV	CTGCTCAAGGCCTGAGACTT
Suppl Figures 5A and 7B	
Globin intron 1 FW	TATCAAGGTTACAAGACAGGTTTAAGGAG
Globin intron 1 RV	ACCACCAGCAGCCTAAGGGTG
